# Urinary Incontinence due to Overactive Detrusor Muscle: A Rare Side Effect of Venlafaxine

**DOI:** 10.1155/2015/690931

**Published:** 2015-09-27

**Authors:** Vithyalakshmi Selvaraj, Palanikumar Gunasekar, Suneel Kumar, Imad Alsakaf

**Affiliations:** ^1^Department of Psychiatry, Creighton University School of Medicine, Omaha, NE 68131, USA; ^2^Department of Biomedical Sciences, Creighton University School of Medicine, CRISS II Room 374, 2500 California Plaza, Omaha, NE 68178, USA

## Abstract

We report a case of reemergence of urinary incontinence (UI) in a patient with benign prostatic hyperplasia (BPH) after starting treatment with venlafaxine who was stabilized on tamsulosin and finasteride for about 6 years. A 66-year-old Caucasian male with prior history of major depressive disorder developed UI within a week of starting venlafaxine 75 mg per day. He described symptoms in the form of involuntary leakage of urine both during the day and at night. His symptoms of UI resolved after stopping the venlafaxine. To the best of our knowledge, there are only four case reports of venlafaxine induced urinary incontinence which have been published.

## 1. Introduction

Venlafaxine is a serotonin-norepinephrine reuptake inhibitor (SNRI) and antidepressant approved by Food and Drug Administration for the treatment of major depression, generalized anxiety disorder, social anxiety disorder, and panic disorder.

We report a case of reemergence of urinary incontinence in a patient with benign prostatic hyperplasia (BPH) after initiating treatment with venlafaxine, who was stabilized on tamsulosin and finasteride for 6 years. To the best of our knowledge, there are only four published case reports of venlafaxine induced urinary incontinence (UI) [1–4].

## 2. Case Presentation

A 66-year-old Caucasian male, has a history of major depressive disorder for which he was started on venlafaxine 75 mg per day, which was titrated to 225 mg/day over a period of three weeks, for his low mood and anxiety. He was diagnosed with BPH (benign prostatic hyperplasia) and struggled with urinary frequency, urgency, urinary incontinence, nocturia, hesitancy, and dribbling of urine. He was prescribed tamsulosin 0.4 mg QDay and finasteride 5 mg Qday. The patient did not have any complaints of BPH and his urinary symptoms were completely resolved for about 6 years. The patient had developed new onset UI within a week of starting venlafaxine. He described his UI in the form of involuntary leakage of urine both during the day and at night.

His past medical history is significant for asbestosis, obstructive sleep apnea, hypertension, coronary artery disease, hyperlipidemia, peripheral neuropathy, arthritis, hiatal hernia, benign prostate hyperplasia, and chronic low back pain. He is allergic to sulfa, meperidine, and felodipine. He was treated with CPAP (continuous positive airway pressure) for his sleep apnea. His other medications were citalopram 80 mg QAm, buspirone 15 mg BID, and mirtazapine 7.5 mg QHS, acetaminophen 325 mg QID, aspirin 81 mg QDay, clonazepam 0.5 mg QDay, docusate 100 mg BID, furosemide 20 mg BID, HCTZ 50 mg Qday, triamterene 75 mg QDay, gabapentin 300 mg TID, morphine 15 mg QID, omeprazole 20 mg BID, propranolol 60 mg QDay, sennosides 8.6 mg BID, and simvastatin 40 mg QDay.

His urine analysis, urine culture, and blood tests including complete blood count and comprehensive metabolic panel were within normal limits which ruled out infectious and metabolic causes for his urinary incontinence. It was decided to discontinue the venlafaxine following which his UI improved. The temporal relationship of the urinary incontinence to the initiation of venlafaxine and the resolution of his UI with the discontinuation of venlafaxine supports our inference that the UI was induced by venlafaxine.

## 3. Discussion

**Figure 1:**
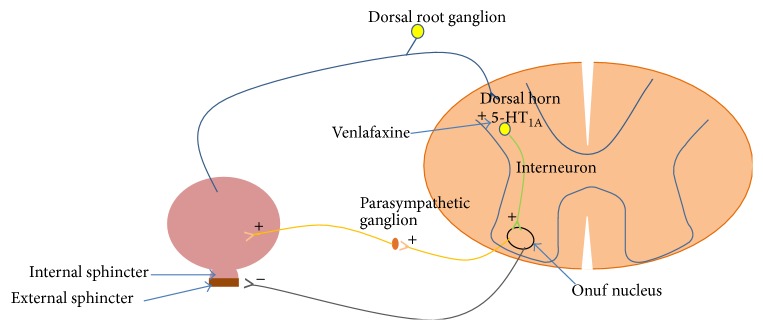


Inghilleri et al. studied the effects of venlafaxine on patients with spinal cord lesion. It was hypothesized that venlafaxine acts at the spinal cord level (Figure 1) where it modulates the detrusor muscle contraction possibly by 5-HT_1A_ receptor activation directly or indirectly on alpha1-adrenoreceptor, causing decrease in detrusor sphincter dyssynergia (DSD) [5]. Tonini et al. also support this hypothesis that activation of 5-HT_1A_ receptor induces detrusor muscle contraction [6].

Another possible mechanism of action for UI is the indirect potentiation of cholinergic neurotransmission in the detrusor muscle of bladder. This is induced by serotonin activation of 5-HT_4_ receptors, which further increases the bladder voiding efficiency resulting in urinary incontinence especially in conditions such as BPH, in this patient [6, 7]. His other medications, citalopram, mirtazapine, buspirone, gabapentin, furosemide, and clonazepam, may have an action on bladder function. However, in this case, it does not seem to be a factor as he was maintained on these medications for few years, and his urinary symptoms resolved after stopping the venlafaxine. Another possible explanation is that venlafaxine level would be increased with medications inhibiting CYP2D6 such as citalopram in this patient. Clinicians should be aware of these rare adverse side effects while prescribing venlafaxine. Interestingly, there is one case report where venlafaxine is used to treat stress incontinence [8]. However, the mechanism of this effect is unclear. Further research is needed in understanding the influence of SNRIs in urinary bladder-sphincter physiology.

## References

[1] Votolato N. A., Stern S., Caputo R. M. (2000). Serotonergic antidepressants and urinary incontinence. *International Urogynecology Journal and Pelvic Floor Dysfunction*.

[2] Polimeni G., Salvo F., Cutroneo P. (2005). Venlafaxine-induced urinary incontinence resolved after switching to sertraline. *Clinical Neuropharmacology*.

[3] Cavanaugh G. L., Martin R. E., Stenson M. A., Robinson D. D. (1997). Venlafaxine and urinary incontinence: possible association. *The Annals of Pharmacotherapy*.

[4] Hansen L. K. (2004). Venlafaxine-induced increase in urinary frequency in 3 women. *Journal of Clinical Psychiatry*.

[5] Inghilleri M., Conte A., Frasca V. (2005). Venlafaxine and bladder function. *Clinical Neuropharmacology*.

[6] Tonini M., Messori E., Franceschetti G. P. (1994). Characterization of the 5-HT receptor potentiating neuromuscular cholinergic transmission in strips of human isolated detrusor muscle. *British Journal of Pharmacology*.

[7] Movig K. L. L., Leufkens H. G. M., Belitser S. V., Lenderink A. W., Egberts A. C. G. (2002). Selective serotonin reuptake inhibitor-induced urinary incontinence. *Pharmacoepidemiology and Drug Safety*.

[8] Erdinc A., Gurates B., Celik H., Polat A., Kumru S., Simsek M. (2009). The efficacy of venlafaxine in the treatment of women with stress urinary incontinence. *Archives of Gynecology and Obstetrics*.

